# Neighborhood Characteristics and Cardiovascular Risk among Older People in Japan: Findings from the JAGES Project

**DOI:** 10.1371/journal.pone.0164525

**Published:** 2016-10-07

**Authors:** Yosuke Inoue, Andrew Stickley, Aki Yazawa, Kokoro Shirai, Airi Amemiya, Naoki Kondo, Katsunori Kondo, Toshiyuki Ojima, Masamichi Hanazato, Norimichi Suzuki, Takeo Fujiwara

**Affiliations:** 1 Department of Human Ecology, Graduate School of Medicine, The University of Tokyo, Bunkyo-ku, Tokyo, Japan; 2 Stockholm Center for Health and Social Change (Scohost), Södertörn University, Huddinge, Sweden; 3 Department of Human Sciences, School of Law and Letters, University of the Ryukyus, Nakagami-gun, Okinawa, Japan; 4 Department of Social Medicine, National Research Institute for Child Health and Development, Setagaya-ku, Tokyo, Japan; 5 Department of Health and Social Behavior, School of Public Health, The University of Tokyo, Bunkyo-ku, Tokyo, Japan; 6 Department of Health Education and Health Sociology, School of Public Health, The University of Tokyo, Bunkyo-ku, Tokyo, Japan; 7 Center for Preventive Medical Sciences, Chiba University, Chiba City, Chiba, Japan; 8 Center for Well-being and Society, Nihon Fukushi University, Nagoya City, Aichi, Japan; 9 Department of Gerontological Evaluation, Center for Gerontology and Social Science, National Center for Geriatrics and Gerontology, Obu City, Aichi, Japan; 10 School of Medicine, Hamamatsu University School of Medicine, Hamamatsu City, Shizuoka, Japan; 11 Department of Global Health Promotion, Tokyo Medical and Dental University, Bunkyo-ku, Tokyo, Japan; JAPAN

## Abstract

Previous studies have found an association between neighborhood characteristics (i.e., aspects of the physical and social environment) and the incidence of cardiovascular disease (CVD) and elevated CVD risk. This study investigated the relationship between neighborhood characteristics and CVD risk among older people in Japan where research on this association is scarce. Data came from the Japan Gerontological Evaluation Study project; questionnaire data collected from 3,810 people aged 65 years or older living in 20 primary school districts in Aichi prefecture, Japan, was linked to a computed composite CVD risk score based on biomarker data (i.e., hemoglobin A1c, systolic blood pressure, diastolic blood pressure, high-density lipoprotein cholesterol, low-density lipoprotein cholesterol, and estimated glomerular filtration rate). A sex-stratified multilevel linear regression analysis revealed that for male participants, living in neighborhoods with a higher perceived occurrence of traffic accidents and reduced personal safety was associated with an elevated CVD risk (coefficient = 1.08 per interquartile range increase, 95% confidence interval [CI] = 0.30 to 1.86) whereas males living in neighborhoods with a higher perceived proximity of exercise facilities had a lower risk (coefficient = −1.00, 95% CI = −1.78 to −0.21). For females, there was no statistically significant association between neighborhood characteristics and CVD risk. This study suggests that aspects of the neighborhood environment might be important for CVD morbidity and mortality in Japan, particularly among men.

## Introduction

In recent years there has been increasing interest in the role of neighborhood characteristics (i.e., aspects of the physical and social environment) in the emergence of cardiovascular disease (CVD)/CVD risk factors [1, 2] given their potential to influence individual behavioral factors that are associated with CVDs such as dietary habits [3–5], physical activity levels [6] and smoking [7, 8]. Previous studies have focused on a wide range of factors linked to the neighborhood physical environment including access to shops selling fresh fruits and vegetables [1, 2, 9], hilly residential areas [10], physical activity resources [1, 2, 11–13], safety from traffic [11, 12], street connectivity [11, 12, 14] and the degree of greenness [15–18]. Similarly, in terms of the social environment, several factors have been examined such as social cohesion [1, 2, 11], structural social capital [19], social support for pro-physical activity [11] and the perceived risk of crime [14, 20, 21]. In the main, these studies have shown that a favorable neighborhood environment is inversely associated with CVD/CVD risk factors, while detrimental neighborhood features have been linked to a greater CVD risk [16, 17].

Neighborhood environments might affect the cardio-metabolic risk of their residents in two main ways: (a) through the impact they have on the energy balance and (b) as a result of psychological stress [2]. For example, energy consumption or dietary composition is influenced by the availability of different food items in the immediate neighborhood environment. Moreover, residing in communities which have exercise and leisure facilities may be associated with a higher level of physical activity [11–13, 15–18], whereas a high crime rate may result in more time being spent indoors, and reduced physical activity. In addition, the detrimental features of communities (e.g. crime, traffic accidents and high unemployment) can also act as chronic psychological stressors, which can damage health both directly [14, 20, 21] and also indirectly through unhealthy behaviors induced by psychological stress [22–24]. In contrast, community-level social capital (i.e., resources accessed by individuals and groups within a social structure that facilitate cooperation, collective action, and the maintenance of norms [25]), might protect people from the effects of psychological stress as a result of greater social support [26, 27] or by underpinning the emergence of a health-promoting environment (e.g., from increased physical activity associated with participating in community organizations) [28].

Despite an increasing focus on the role of neighborhood characteristics in the emergence of CVD/CVD risk, there are still important research gaps. For example, although most studies have used various predictor variables (e.g., smoking, physical activity, body mass index (BMI), hypertension, fruit and vegetable consumption) as CVD risk factors, few have simultaneously assessed multiple biomarkers which would more accurately represent the overall risk for a future CVD event [1, 11, 20, 29–31]. Further, these associations have still not yet been studied in many parts of the world. For instance, to the best of our knowledge, there is no research on the association between aspects of the physical and social neighborhood environment and CVD risk in Japan, even though CVD is one of the leading causes of death in the country [32].

To address this issue the current study used data from the Japan Gerontological Evaluation Study (JAGES) project, an ongoing epidemiological study that focuses on identifying social determinants of health among Japanese people aged 65 years and older [33], to investigate the association between neighborhood characteristics and CVD risk as evaluated by multiple biomarkers.

## Methods

### Data

The study data were collected through a postal survey undertaken in August 2010 to January 2012 in six municipalities in Chita peninsula, Aichi prefecture, Japan. This was linked to data obtained in the municipality-organized voluntary health check-up (the Tokutei kenkou shinsa: Kenshin) in four of the six municipalities. Of the 20,432 people who participated in the JAGES project in Aichi prefecture, health check-up information was linked for 9,893 (48.4%). Study exclusion criteria included missing information for either: location or residence (at a primary school zone level) (n = 9), weight and height (n = 41), biomarkers (n = 5,530); current smoking status (n = 409). Individuals who had self-reported difficulty in daily living activities were also excluded (n = 293). In addition, one municipality did not provide information on blood pressure. A total of 3,810 participants residing in 20 primary school districts in 3 municipalities were thus included in the subsequent analyses.

The study protocol and questionnaire procedures were approved by the Ethics Committee for Research on Human Subjects at Nihon Fukushi University, Japan (No. 10–05) and the Ethics Committee for Medical Research at the University of Tokyo (No. 10555). Informed consent was assumed with the voluntary return of the questionnaire.

### Dependent variable

CVD risk was evaluated with the Suita Score [34] using information from six biomarkers measured in the health check-up (i.e., hemoglobin A1c (HbA1c), systolic and diastolic blood pressure, low-density (LDL) and high-density (HDL) lipoprotein cholesterol and the estimated glomerular filtration rate (eGFR)) together with age, sex and current smoking status. The Suita Score was developed by the National Cerebral and Cardiovascular Center, Japan, to predict possible future CVD events; it was developed based on the Framingham Risk Score [35], which is known to overestimate the risk of coronary heart disease among the Japanese population [34]. The score can range from 10 to 95 with higher values indicating a higher risk of a future cardiovascular event.

### Independent variables

The questionnaire obtained information on different aspects of the neighborhood environment. Following the lead of previous studies [1, 2], we examined: (1) the proximity of shops selling fresh fruits and vegetables; (2) the proximity of exercise facilities (i.e., places that are suitable for exercise or walking); (3) the presence of slopes or stairs in the neighborhood; (4) social capital; and (5) personal safety and the risk of traffic accidents.

### Physical environment

For the first three above-mentioned variables (1–3), participants were asked about the environment within 1 km of their residential location (i.e., about shops or facilities that sell fresh fruits and vegetables, parks or streets which are suitable for sports activities or walking, and places where they find it difficult to walk because of environmental obstacles such as slopes or stairs). They used one of five answer options to describe the extent to which these factors were present in their immediate environment (i.e., greatly, to some extent, a few, none and don’t know). For the current study we calculated the proportion of those who answered either “greatly” or “to some extent” by primary school district.

These three neighborhood environmental features were also evaluated by geographic information system (GIS) analysis; the number of shops which purportedly sold fresh food (such as fruits, vegetables, meat and fish), the number of parks (i.e., exercise facilities), and the hilliness of the neighborhood were calculated for each primary school district. Information on the number of grocery stores at a 500-meter resolution was available from the 2007 Commerce Census conducted by the Ministry of Economy, Trade and Industry; in this study, the number of grocery stores was calculated for each school district, assuming that all of the stores were located in the centroids of each geographical unit (i.e., 500m×500m). A grocery store was defined as either a department store, general merchandise store, specialized supermarket, or daily commodities store. The average park count was calculated using National Land Numerical Information City Park Data (as of 2011) from the Ministry of Land, Infrastructure, Transport and Tourism (MLIT), Japan. The average hilliness of each neighborhood i.e. the land slope was calculated based on the Elevation, Degree of Slope 5th Mesh Data (as of 2011) which was also obtained from MLIT and was based on the Digital Map 50 m Grid (Elevation). ArcGIS 10.1 software was used for all spatial calculations.

### Social environment

Community-level social capital was operationalized in two distinct forms. Cognitive social capital, which includes norms, values, attitudes and beliefs [36], was measured with the following three questions, “generally speaking, do you trust people in your community?”, “do people in your community try to be helpful to others?” and “how attached do you feel to your community?” Each question had five answer options (i.e., greatly, to some extent, cannot say, not very much, and not at all). The proportion of those who answered greatly or to some extent for each question was calculated, standardized to a mean of 0 and standard deviation of 1 and then averaged to create a composite score for each primary school district. Structural social capital, which is usually operationalized as the extent and intensity of associational links and activity in society [36], was measured with three questions on social participation (i.e., participating in volunteer groups, sports organizations and hobby groups at least once a month). The proportion of those who participated in each activity was calculated, standardized and then averaged to create a composite score. Perceived personal safety and the risk of neighborhood traffic accidents was evaluated with three questions; “Is there any road or intersection which is a high risk for traffic accidents?”, “Is there any place where you think it is dangerous to walk at night?”, and “how unsafe do you feel in your community?” The proportion of those who answered “greatly” or “to some extent” for each question was calculated and standardized to compute a composite score.

### Covariates

Information on participants’ demographic and socioeconomic characteristics was also collected (i.e. age, sex, marital status, educational attainment, employment and household equivalent income). BMI was calculated based on self-reported height and weight. After excluding those whose height or weight was above or below 4 SD from the mean, BMI was divided into three categories: < 18.5; 18.5 to 24.9; ≥ 25.0. Marital status referred to being either married or not married, or was unknown. There were three educational attainment levels based on the number of years spent in school, ≤ 9 years; 10–12 years; and ≥ 13 years. For employment status respondents were categorized as being either employed; retired and not employed; had never worked; or had missing data. Annual household equivalent income was calculated by dividing the total household income by the square root of the number of household members [37] and was divided into three categories: low (less than 2 million yen); middle (2 to 4 million yen); and high (more than 4 million yen). Mental health i.e., the presence of depression, was assessed using the 15-item Geriatric Depression Scale (GDS), a self-administered questionnaire. Participants were categorized into three groups i.e., non-depressed (a score of 0–4); mild depression (5–9); and severe depression (10–15) [38].

Lifestyle information was collected on (1) the frequency of consuming fruit and vegetables, (2) alcohol consumption, (3) current cigarette smoking, (4) time spent in daily walking and (5) the frequency of meeting friends. The frequency of fruit and vegetable consumption was assessed with the question, “How often did you eat fruit and vegetables in the previous month?” Responses were grouped into four categories, ≥ twice a day; once a day; four to six times a week; ≤ two to three times a week. Alcohol consumption was categorized with the answer options yes, no, and used to drink. Smoking status was assessed by the question, “Do you smoke?” with four possible answers, never; quit smoking more than 5 years ago; quit smoking 5 or less years ago; and currently smoke. To assess time spent in daily walking respondents were asked, “How long do you walk for each day on average?” with four response options, < 30 minutes; 30–59 minutes; 60–89 minutes; and ≥ 90 minutes. Information on the frequency of meeting friends was obtained with the question “How often do you meet friends or acquaintances?” with answers divided into two categories, ≥ once a week; and < once a week. Neighborhood socioeconomic status (SES) was evaluated by calculating the land price of each primary school zone for the year 2010, with information which was published by MLIT [39].

### Statistical analysis

Following the lead of a previous study which investigated the association between neighborhood SES and the Framingham Score [40], a multilevel linear regression analysis was conducted to investigate the association between neighborhood characteristics and the Suita Score as a continuous variable. Sex-specific analyses were undertaken as previous research has indicated that not only do male and female lifestyles differ in Japan [32, 41] but that the way the sexes interact with their surrounding neighborhood may differ [42]. For the multilevel analysis we defined individual participants and primary school districts as Level 1 and Level 2, respectively. To ensure comparability the nine neighborhood characteristics that were the focus of this study were standardized (i.e. divided by their inter-quartile range [IQR] values) and examined separately, with models being adjusted sequentially [2]. Model 1 adjusted for individual socio-demographic variables (i.e., age in years, age-squared, sex, BMI, marital status, educational status, employment status, equivalent household income), depression and neighborhood SES; Model 2 further adjusted for individual behavioral variables (i.e., time spent walking, fruit and vegetable consumption, alcohol consumption, smoking and frequency of meeting friends). To maximize statistical power, if information was missing for any of the covariates a “missing” category was created and included in the analysis. Additional analyses showed that the overall results did not change when the participants with missing information were excluded. When investigating the effect of neighborhood cognitive and structural social capital, we also included individual-level cognitive (i.e., trust, attachment and reciprocity) and structural (i.e., participation in sports organizations, volunteer groups and hobby clubs once a month or more frequently) social capital measures as covariates, respectively.

To examine the robustness of the results, we also performed a sensitivity analysis, where the analysis was restricted to those who had resided in the primary school districts for 10 years or longer while adjusting for the same covariates included in Model 2, as it is possible that the effects of the neighborhood environment may be cumulative and operate over a longer period of time. We chose 10 years as a cut-off principally because the Suita Score and several similar scores usually calculate the 10-year risk of CVD, which means 10 years is thought of as being a long enough period of time to differentiate those who are at risk and those who are not in terms of developing CVD.

Stata 13.0 (Stata Corp, College Station, TX) was used to conduct the statistical analyses. Results are shown in the form of coefficients with 95% confidence intervals (CI). The level of statistical significance was p < 0.05.

## Results

Descriptive statistics of the study participants are presented in Table 1. Participants’ average age was 71.3 years and 52.6% were female. More than three quarters of the participants were married, while just under half of them (47.8%) had ≤ 9 years education, 36.1% had 10–12 years of education while 12.4% had obtained a further education (≥ 13 years). Most households (42.2%) were in the lowest income category of less than 2 million yen. Just under half of the participants (46.4%) consumed vegetables twice a day or more often with 40% of them reporting that they drink alcohol although this proportion was more than three times higher among male participants (63.1% vs. 19.1%). A sex-based imbalance was also observed for current smoking with 19.0% of the male participants smoking compared to only 2.4% of females. Nearly one-third (32.7%) of the participants walked for at least one hour every day, while 75.3% of them met with friends one or more times a week. There was no statistically significant difference by sex in HbA1c, systolic blood pressure or eGFR, while diastolic blood pressure was higher among males and LDL and HDL cholesterols were statistically higher among females. The total Suita Score was significantly higher among male participants.

**Table 1:** Characteristics of the study participants in Chita peninsular, Aichi Prefecture, Japan in 2010 (n = 3,810).

	Total	Men	Women	p-value
	(n = 3,810)	(n = 1,805)	(n = 2,005)	
	*Mean [SD] / n (%)*			* *
Age (in years)	71.3 [5.0]	71.2 [4.8]	71.4 [5.2]	0.28
Marital status				
Married	2,935 (77.0)	1,586 (87.9)	1,349 (67.3)	< 0.001
Not married	748 (19.6)	164 (9.0)	586 (29.2)	
Other	127 (3.3)	57 (3.2)	70 (3.5)	
Educational attainment				
≤ 9 years	1,822 (47.8)	782 (43.3)	1,040 (51.9)	< 0.001
10–12 years	1,376 (36.1)	678 (37.6)	698 (34.8)	
≥ 13 years	474 (12.4)	282 (15.6)	192 (9.6)	
Missing	138 (3.6)	63 (3.5)	75 (3.7)	
Annual equivalent household income (yen)				
< 2 million	1,607 (42.2)	765 (42.4)	842 (42.0)	< 0.001
2–4 million	1,380 (36.2)	732 (40.6)	648 (32.3)	
> 4 million	234 (6.1)	112 (6.2)	122 (6.1)	
Missing	589 (15.5)	196 (10.9)	393 (19.6)	
Occupational status				
Currently employed	701 (18.4)	411 (22.8)	290 (14.3)	< 0.001
Retired	2,314 (60.7)	1,228 (68.0)	1,086 (54.2)	
Unemployed	374 (9.8)	64 (3.6)	310 (15.5)	
Missing	421 (11.1)	102 (5.7)	319 (15.9)	
BMI (kg/m^2^)				
Lean (< 18.5)	195 (5.1)	52 (2.9)	143 (7.1)	0.001
Normal (18.5–24.9)	2,704 (71.0)	1,300 (72.0)	1,404 (70.0)	
Overweight (25–29.9)	827 (21.7)	426 (23.6)	401 (20.0)	
Obese (> 30)	84 (2.2)	27 (1.5)	57 (2.8)	
GDS				
Not depressed (0–4)	2,260 (59.3)	1,030 (57.1)	1,230 (61.4)	0.01
Mild depression (5–9)	720 (18.9)	346 (19.2)	374 (18.7)	
Depression (10–15)	196 (5.1)	93 (5.2)	103 (5.1)	
Missing	634 (16.6)	336 (18.6)	298 (14.9)	
Vegetable intake				
> twice/day	1,767 (46.4)	685 (38.0)	1,082 (54.0)	< 0.001
Once/day	1,252 (32.9)	638 (35.4)	614 (30.6)	
4–6 times/week	444 (11.7)	275 (15.2)	169 (8.4)	
2–3 times/week or less	304 (8.0)	195 (10.8)	109 (5.4)	
Missing	43 (1.1)	12 (0.7)	31 (1.6)	
Alcohol consumption				
Drink	1,521 (39.9)	1,138 (63.1)	383 (19.1)	< 0.001
Don’t drink	2,249 (59.0)	656 (36.3)	1,593 (79.5)	
Missing	40 (1.1)	11 (0.6)	29 (1.5)	
Cigarette smoking				
Never	2,310 (60.6)	448 (24.8)	1,862 (92.9)	< 0.001
Quit	1,109 (29.1)	1,014 (56.2)	95 (4.7)	
Smoke	391 (10.3)	343 (19.0)	48 (2.4)	
Walking hours				
< 30 min	1,070 (28.1)	458 (25.4)	612 (30.5)	< 0.001
30–59 min	1,297 (34.0)	614 (34.0)	683 (34.1)	
60–89 min	634 (16.6)	329 (18.2)	305 (15.2)	
≥ 90 min	612 (16.1)	335 (18.6)	277 (13.8)	
Missing	197 (5.2)	69 (3.8)	128 (6.4)	
Meeting friends				
< once/week	745 (19.6)	496 (27.5)	249 (12.4)	< 0.001
≥ once/week	2,869 (75.3)	1,233 (68.3)	1,636 (81.6)	
Missing	196 (5.1)	76 (4.2)	120 (6.0)	
HbA_1c_ (%)	5.6 [0.7]	5.6 [0.8]	5.5 [0.6]	0.29
Systolic blood pressure (mmHg)	131.7 [16.5]	131.9 [16.4]	131.5 [16.5]	0.56
Diastolic blood pressure (mmHg)	73.7 [10.2]	74.8 [10.5]	72.8 [9.9]	< 0.001
LDL cholesterol (mg/dL)	119.7 [28.9]	114.6 [29.0]	124.2 [28.0]	< 0.001
HDL cholesterol (mg/dL)	58.9 [15.6]	54.7 [15.0]	62.6 [15.2]	< 0.001
eGFR(mL/min/1.73 m^2^)	68.9 [13.9]	68.3 [14.1]	69.4 [13.6]	0.17
Suita Score	49.9 [7.5]	54.2 [6.7]	46.1 [5.8]	< 0.001

Details of the neighborhood characteristics evaluated at a primary school district level are presented in Table 2. Seventy-five percent of the participants in each school district answered that there were shops selling fresh fruit and vegetables in the vicinity (76.4, IQR = 69.3 to 79.9), while the proportion was almost identical for the perceived proximity of exercise facilities (76.1, IQR = 68.7 to 83.8). A much lower proportion of respondents perceived barriers to walking in their immediate environment (43.0, IQR = 34.2 to 53.9). A GIS-based analysis revealed there were 5.4 shops (median, IQR = 3.0 to 12.6) per square kilometer (km^2^) that supposedly sold fresh fruit and vegetables, while there were 0.88 (median, IQR = 0.19 to 2.02) parks per km^2^. The median value of the neighborhood slopes (a measure of hilliness) was 3.17 degrees (IQR = 2.30 to 4.44). The school district-level indices of cognitive and structural social capital were 0.03 (IQR = −0.38 to 0.23) and 0.00 (IQR = −0.25 to 0.61). The median value for perceived personal safety and risk of traffic accidents was 0.25 (IQR = −0.57 to 0.56). The median land price was 64,362.5 yen (IQR = 42,550 to 82,375) per square meter.

**Table 2:** Basic attributes of the neighborhood environment in the study locations in Chita peninsular, Aichi Prefecture, Japan (n = 20).

Attributes	Median	IQR
1. Proximity of shops selling fresh vegetables and fruits		
1a. Perceived (%)	76.4	69.3, 79.9
1b. GIS-based (per km^2^)	5.4	3.0, 12.6
2. Proximity of exercise facilities		
2a. Perceived (%)	76.1	68.7, 83.8
2b. GIS-based (per km^2^)	0.88	0.19, 2.02
3. Walking environment in the neighborhood		
3a. Perceived (%)	43.0	34.2, 53.9
3b. GIS-based hilliness (degree)	3.17	2.30, 4.44
4. Social capital		
4a. Cognitive	0.03	−0.38, 0.23
4b. Structural	0.00	−0.25, 0.61
5. Personal safety and risk of traffic accidents		
5a. Perceived	0.25	−0.57, 0.56
Neighborhood SES		
Land price (yen per m^2^)	64,362.5	42,550, 82,375

In the multilevel linear regression analysis male participants who lived in neighborhoods with a higher perceived proximity of exercise facilities had a lower CVD risk (Table 3). Each IQR increase in the index of the perceived proximity of exercise facilities resulted in a 0.77 (95% confidence interval [CI] = −1.48 to −0.07) reduction in the Suita Score in Model 1. Specifically, those who resided in communities with a greater number of perceived exercise facilities (i.e., the top 25%) had a 0.77 lower score compared with those in communities with fewer perceived exercise facilities (i.e., the bottom 25%). This remained statistically significant even after adjusting for individual behavioral factors (coefficient = −1.00, 95% CI = −1.78 to −0.21). In contrast, living in a neighborhood where the perceived risk of traffic accidents and reduced personal safety was greater was associated with a higher CVD risk (i.e., coefficient = 0.81, 95% CI = 0.11 to 1.52) among men, which specifically meant that those in communities with a higher perceived risk of traffic accidents and reduced personal safety (i.e., the top 25%) had 0.81 points higher score compared with those residing in communities with a lower perception; this also remained statistically significant after adjusting for individual behavioral factors in Model 2 (coefficient = 1.08, 95% CI = 0.30 to 1.86). Among the female participants, there was no statistically significant association between any of the neighborhood characteristics and CVD risk.

**Table 3:** Multilevel linear regression analysis examining the association between neighborhood characteristics and cardiovascular risk among older people living in Chita peninsula, Aichi Prefecture, Japan.

	Male participants (n = 1,805)				Female participants (n = 2,005)			
	Model 1		Model 2		Model 1		Model 2	
	coef.	95% CI	coef.	95% CI	coef.	95% CI	coef.	95% CI
1. Proximity of shops selling fresh fruits and vegetables								
1a. Perceived	−0.02	−0.48, 0.45	−0.06	−0.63, 0.50	−0.15	−0.67, 0.37	−0.15	−0.66, 0.36
1b. GIS-based	0.12	−0.69, 0.92	−0.04	−0.95, 0.87	0.14	−0.63, 0.92	0.07	−0.70, 0.83
2. Proximity of exercise facilities								
2a. Perceived	−0.77	−1.48, −0.07*	−1.00	−1.78, −0.21*	−0.16	−0.96, 0.64	−0.22	−1.01, 0.56
2b. GIS-based	−0.14	−0.59, 0.30	−0.09	−0.61, 0.43	0.15	−0.34, 0.64	0.10	−0.38, 0.59
3. Walking environment in the neighborhood								
3a. Perceived	−0.58	−1.25, 0.08†	−0.62	−1.39, 0.14	−0.30	−0.98, 0.38	−0.31	−0.98, 0.35
3b. GIS-based	−0.21	−0.76, 0.34	−0.16	−0.83, 0.51	0.08	−0.55, 0.71	0.09	−0.54, 0.71
4. Social Capital								
4a. Cognitive	−0.16	−0.57, 0.25	−0.26	−0.71, 0.19	−0.14	−0.55, 0.27	−0.17	−0.57, 0.23
4b. Structural	−0.27	−0.94, 0.41	−0.02	−0.77, 0.74	0.07	−0.61, 0.74	0.08	−0.59, 0.75
5. Personal safety and risk of traffic accidents								
5a. Perceived	0.81	0.11, 1.52*	1.08	0.30, 1.86**	0.40	−0.37, 1.17	0.44	−0.31, 1.19

**: p < 0.01

*: p < 0.05

†: p < 0.10

Model 1 adjusted for basic individual socio-demographic variables (i.e., age in years, age-squared, sex, body mass index, marital status, educational status, employment status, equivalent household income and depression) and neighborhood SES. When investigating the effect of neighborhood cognitive and structural social capital (4a and 4b), we also included individual-level cognitive and structural social capital measures as covariates, respectively.

Model 2 further adjusted for individual behavioral variables (i.e., time spent walking, fruit and vegetable consumption, alcohol consumption, smoking and frequency of meeting friends).

When the analysis was restricted to those participants who had resided in their current residential location for 10 years or longer, the associations observed among the male participants were generally more pronounced (Table 4), while there was no significant association between any of the neighborhood characteristics and CVD risk among the female participants.

**Table 4:** Sensitivity analysis restricted to those participants who had resided in their current location for ≥ 10 years in Chita peninsula, Aichi Prefecture, Japan.

	Male participants (n = 1,692)				Female participants (n = 1,899)			
	Model 1		Model 2		Model 1		Model 2	
	coef.	95% CI	coef.	95% CI	coef.	95% CI	coef.	95% CI
1. Proximity of shops selling fresh fruits and vegetables								
1a. Perceived	−0.11	−0.58, 0.37	−0.16	−0.72, 0.41	−0.14	−0.64, 0.37	−0.12	−0.63, 0.38
1b. GIS-based	0.07	−0.74, 0.89	−0.07	−0.99, 0.85	0.13	−0.64, 0.90	0.05	−0.70, 0.81
2. Proximity of exercise facilities								
2a. Perceived	−0.84	−1.56, −0.13*	−1.07	−1.86, −0.28**	−0.18	−0.96, 0.61	−0.22	−0.99, 0.55
2b. GIS-based	−0.14	−0.59, 0.31	−0.11	−0.62, 0.41	0.13	−0.35, 0.61	0.09	−0.39, 0.56
3. Walking environment in the neighborhood								
3a. Perceived	−0.53	−1.22, 0.16	−0.57	−1.35, 0.21	−0.27	−0.94, 0.41	−0.28	−0.94, 0.39
3b. GIS-based	−0.29	−0.83, 0.24	−0.27	−0.92, 0.38	0.04	−0.58, 0.66	0.05	−0.56, 0.66
4. Social Capital								
4a. Cognitive	−0.20	−0.60, 0.21	−0.32	−0.77, 0.12	−0.14	−0.54, 0.26	−0.15	−0.54, 0.25
4b. Structural	−0.25	−0.94, 0.43	−0.01	−0.77, 0.75	0.07	−0.60, 0.75	0.10	−0.56, 0.76
5. Personal safety and risk of traffic accidents								
5a. Perceived	0.91	0.23, 1.60**	1.18	0.43, 1.94**		0.34	−0.42, 1.11	0.39	−0.36, 1.14

**: p < 0.01

*: p < 0.05

Models were adjusted for basic individual socio-demographic variables (i.e., age in years, age-squared, sex, body mass index, marital status, educational status, employment status, equivalent household income and depression) and neighborhood SES and individual behavioral variables (i.e., time spent walking, fruit and vegetable consumption, alcohol consumption, smoking and frequency of meeting friends). When investigating the effect of neighborhood cognitive and structural social capital (4a and 4b), we also included individual-level cognitive and structural social capital measures as covariates, respectively.

## Discussion

### Summary of the study’s main findings

The present study revealed that for males aged 65 years or older, living in a neighborhood with a higher perceived proximity to exercise facilities was associated with a reduced CVD risk, while those who lived in neighborhoods where traffic accidents and reduced safety were perceived as being more common had a higher CVD risk. Among female participants, no associations were observed between neighborhood characteristics and CVD risk. An additional analysis that focused only on those participants who had resided in their current residential location for at least 10 years produced the same results although the associations seen among men were more pronounced.

### Mechanisms linking neighborhood characteristics and cardiovascular risk

To the best of our knowledge, no previous study has examined the association between the perceived proximity of exercise facilities and CVD risk, although research on other health outcomes has produced similar findings to the associations seen among men in this study. For example, Auchincloss et al. [43] found that neighborhood physical activity resources were associated with a lower hazard ratio for diabetes (0.65) in the U.S. Mathis et al. [44] investigated the association between BMI and local recreational facilities and showed that obese older adults had a significantly lower probability of having a park in their neighborhood. Previous research has also highlighted the ways in which such an environment might be beneficial for health. For instance, an earlier study showed that people who live in areas where there are more parks are more likely to walk for at least 60 minutes per week, compared to those who live in areas with fewer parks [12]. In addition, in Canada, park features (the number of facilities in a park such as open spaces, paths, playgrounds, wooded areas, and unpaved trails) were a significant predictor of physical activity [13]. In England, the reported frequency of green space use among people declined with increasing distance whereas those individuals who lived closest to different types of green space tended to achieve the recommended level of physical activity [15]. The results of the current study suggest that such health-promoting facilities may also have the potential to reduce CVD risk in Japan. Future studies should obtain more detailed objective information about how respondents interact with the specific features (e.g. parks) of the surrounding environment to elucidate more precisely how these neighborhood characteristics might influence CVD risk.

The association between perceived neighborhood safety, risk of traffic accidents and CVD risk observed among men accords with the findings from earlier studies that have used the same/similar neighborhood predictor variables. For example, in Sweden high levels of neighborhood violent crime have been associated with coronary heart disease (CHD) (i.e., a first hospitalization for a fatal or non-fatal CHD event) [20]. Similarly, neighborhood safety (defined as being able to walk safely during the day and at night, crime and violence) has also been linked to the cardiovascular health (CVH) score, a concept recently introduced by the American Heart Association [1]. The perceived risk of traffic accidents and reduced personal safety have also been associated with other health indicators such as obesity [21, 44], mental well-being [45–47] and physical functioning [47]. Living in fear might result in more time being spent indoors, lower levels of physical activity, and higher levels of psychological stress, all of which have the possibility to impact on CVD risk. It should also be emphasized that we observed this association with perceived safety in a setting (Japan) where crime is not as common as in other countries [48]. This highlights the importance of perceived safety on CVD risk and the need for future research to determine the factors underlying this association more precisely (e.g. what level of crime has an effect, does personal victimization explain this association etc.).

Although previous studies showed that living in a favorable food environment was associated with a lower CVD risk, in the current study we did not observe this association. For example, Unger et al. [1] found that the presence of more healthily provisioned neighborhood food stores was positively associated with CVH scores in the U.S. One possible reason for the null finding in the current study is that the variation in the food environment was not large enough to be mirrored in the CVD risk. Another possibility comes from the fact that we did not consider the availability of fruit and vegetables from other sources such as mobile vendors or catering services or whether participants grew their own produce.

The association between the neighborhood walking environment (e.g., the perceived presence of slopes and stairs and a GIS measure of neighborhood ‘hilliness’) and CVD risk or health in general has not been extensively studied. An earlier study undertaken in Australia nonetheless showed that the odds of self-reported diabetes were lower among those who lived in neighborhoods with steeper slope levels [10]. As neighborhoods were hillier on average in our study (3.2 degrees) than the Australian study (3.3%, equivalent to 1.9 degrees), it would have been reasonable to expect that there would have also been an association with CVD risk in this study on the basis that hilliness is likely to be associated with an increase in energy expenditure; however, hilly environments might also prevent people from undertaking physical activity (especially those who are older).

To date, research on the association between neighborhood social capital and CVD risk has produced conflicting results. Unger et al. [1] showed for example, that social capital (termed as social cohesion in their study) was not associated with the CVH score. In contrast, Scheffler et al. [19] found that a one-standard deviation increase in a social capital index (i.e., the Petris Social Capital Index) was significantly associated with a decreased recurrence of acute coronary syndrome, and that this was observed among those living in areas with lower SES. In addition, one component of neighborhood cognitive social capital i.e., a lack of fairness, has also been linked with decreased systolic blood pressure [49]. In the present study we did not find an association when using the Suita Score. It is uncertain what underlies this difference, although as Unger et al. [1] pointed out, it is possible that the mechanisms linking social capital and CVD risk might operate more at the individual level. Indeed, in this study, those men who participated in volunteer groups had a lower CVD risk (coefficient = −1.35, p = 0.003; data not shown in table), while Yazawa et al. [50] who also used JAGES data found that individual-level social participation was inversely associated with hypertension.

### The sex difference in the association between neighborhood characteristics and CVD risk

Earlier research showed that there was a sex difference in the effects of the neighborhood environment on cardiovascular risk. However, it also highlighted that there was a more pronounced association among female participants, which is opposite to the finding of the current study. For example, the effect of inequity on CHD incidence and case fatality as measured by neighborhood deprivation has been found to be slightly stronger for women than men [51]. A sex difference has also been seen for other health outcomes. In a study undertaken in England [47], multiple aspects of the residential environment (e.g., trust, integration into the wider society, a left-wing political climate, the physical quality of the residential environment, and the unemployment rate) had a larger effect for females than males in terms of self-rated health. Inoue et al. [52] also found that females were more affected by neighborhood socioeconomic characteristics (i.e., differences in the Gini coefficient) than men in terms of C-reactive protein concentration in rural China. Furthermore, while studying the French population, Vallée et al. [53] suggested that females might be more influenced by the place where they live since they tend to spend more time in their community surroundings.

One possible reason for these conflicting findings might relate to differences in the age range of the study participants. Our participants were aged 65 years or older. Yasunaga et al. [54] showed that among old people living in another location in Japan, the age-related reduction in habitual physical activity was reflected in a reduction in activity ≥ 3 metabolic equivalents (METs; multiples of resting metabolic rate) among men while for women it was < 3 METs, where 3 METs is the moderate physical activity intensity level recommended by the World Health Organization to reduce non-communicable diseases [55]. Given this, the sex difference observed in the present study might have resulted from the different degrees to which sex moderated the age-related reduction in environmental physical activity. Another possibility comes from the fact that the mean CVD risk score was lower among females (i.e., females were generally healthier than males); it is possible that it might have been more difficult to detect a neighborhood effect among females, as they were generally healthier irrespective of their surrounding environment.

### Study limitations and future research

This study has several limitations. First, our study population might not have been fully representative of all older persons living in Japan in several respects. For example, as our survey was a postal survey which required a physical response to return the completed questionnaire it is possible that those who had mobility or other serious health problems may have been automatically excluded. Furthermore, as we used data from a non-compulsory health check-up it is possible that our participants might have differed systematically from those who did not undergo the medical examination in terms of such things as their socio-economic or health status or how much they interact with the neighborhood environment–potentially biasing our results. In addition, this study only focused on those who lived in Chita peninsular which limits the generalizability of our findings. Second, the cross-sectional nature of the study precluded the possibility of establishing causal relations. To further elucidate the impact of the neighborhood environment on CVD risk in this setting, future studies should investigate these associations using longitudinal data. Third, we lacked information on potentially important explanatory variables which might have affected both health and participants’ perceptions of the neighborhood environment e.g. information on the purchasing of fresh fruits and vegetables (such as what was purchased and where it was purchased from), consumption of homegrown vegetables, availability and utilization of exercise facilities (not only parks but also other facilities such as recreational centers), incidence of crime or personal experience of victimization. In addition, as information on the number of grocery shops was obtained from the 2007 Commerce Census, it is also possible that the number of shops had changed by the time the study was undertaken (i.e., 2010). Fourth, as the Suita Score has been validated only among people residing in Suita City, Osaka, CVD risk estimation might not have been accurate in this study.

Future research should focus on how to combine different methods of assessing the neighborhood environment (e.g. GIS-based methods and participants’ perceptions) and to determine what underlies any differences in outcomes such as the difference that was observed in the current study, where statistically significant associations were only observed for participant perceptions of the neighborhood environment. In addition, as there has been comparatively little research about the effects of the neighborhood environment on health and well-being among older age groups, this should also be a priority for future research, especially given the ongoing process of population aging in Japan and other countries.

## Conclusion

This study revealed that for older men living in a neighborhood with a higher perceived proximity to exercise facilities was inversely associated with CVD risk, while living in neighborhoods where traffic accidents and reduced safety were perceived as being more common was positively associated with CVD risk. This study suggests that efforts by the Japanese government to create a healthy living environment might be important in combating CVD morbidity and mortality in Japan, particularly among men.
